# An Overview of the Role of Medicinal Plants in Parkinson’s Disease: A Semi-Systematic Review

**DOI:** 10.3390/biomedicines13082008

**Published:** 2025-08-18

**Authors:** Hedie Haxhiu, Malvina Hoxha, Ina Zela, Bruno Zappacosta

**Affiliations:** 1 Department of Pharmacy, Faculty of Pharmacy, University of Granada, 18012 Granada, Spain; hhaxhiu@correo.ugr.es; 2 Department for Chemical-Toxicological and Pharmacological Evaluation of Drugs, Faculty of Pharmacy, Catholic University Our Lady of Good Counsel, 1000 Tirana, Albania; 3 Department of Pharmacy, University of Medicine, 1000 Tirana, Albania; inazela92@gmail.com; 4 Department of Biomedical Sciences, Faculty of Medicine, Catholic University Our Lady of Good Counsel, 1000 Tirana, Albania; b.zappacosta@unizkm.al

**Keywords:** *Curcuma longa*, *Gastrodia elata blume*, *Ginkgo biloba*, *Paeonia alba radix*, *Pueraria lobata*, Parkinson, *Scutellaria baicalensis*, *Withania somnifera*

## Abstract

**Background/Objectives**: Parkinson’s disease (PD) is a complex nervous system disorder characterized by the gradual loss of dopaminergic neurons, leading to disturbances in movement, such as resting tremors, rigidity, bradykinesia, or akinesia; postural issues; and freezing (motor block). Due to the limitations and side effects of current pharmacological treatments, there is a growing interest in investigating the therapeutic potential of medicinal plants. **Methods**: A semi-systematic review was conducted using PubMed, Web of Science, and Scopus as main databases, identifying original research articles, systematic reviews, and relevant preclinical or clinical studies published between January 2000 and December 2024. We selected seven plants primarily for their neuroprotective effects, supported by preclinical and animal data. Only articles in English were included in the study. **Results**: Seventeen articles were included in the study. The results showed that *Curcuma longa*, *Gastrodia elata blume*, *Ginkgo biloba*, *Paeonia alba radix*, *Pueraria lobata*, *Scutellaria baicalensis*, and *Withania somnifera* have a neuroprotective role, capable of slowing down the progression of PD with different mechanisms of action, ranging from restorative properties of neurons. **Conclusions**: Developing new drugs based on the respective herb compounds/extracts and herbal formulas is a promising avenue for complementary therapies for PD. However, further preclinical and clinical studies are required to confirm their safety, efficacy, bioavailability, and dosage.

## 1. Introduction

Parkinson’s disease (PD) is a neurodegenerative disorder, the second most widespread degenerative disease affecting the central nervous system (CNS). According to the World Health Organization (WHO), the prevalence of PD has doubled in the past 25 years. Global estimates in 2019 showed over 8.5 million individuals with PD [1]. The prevalence of PD increases with age, and only about 4% of new cases are discovered before the age of 50. Genetic predisposition, age-related dysfunctions, and environmental factors (such as metal exposure) are known to contribute to PD pathogenesis. Although the mechanism involved in its pathogenesis is not fully understood, the defining feature of the disease is the declining dopaminergic function in the “substantia nigra pars compacta” region, deposit of the Lewi’s bodies, and insoluble cytoplasmic aggregates of α-synuclein [2].

PD’s diagnosis criteria are based on clinical signs related to the four main motor symptoms: bradykinesia, muscle stiffness, tremor, and impaired postural reflexes.

These symptoms derive from the imbalance between the dopaminergic pathway originating in the mesencephalon and the glutamatergic pathways from the cortical, thalamic, and limbic regions. This imbalance reduces cortical input due to insufficient inhibition of D2 dopaminergic receptors [3].

Scientific research has documented that inflammation, oxidative stress, apoptosis, and loss of neurotrophic factors are some of the key factors involved in the pathophysiology of PD. Oxidative stress interferes with dopamine (DA) metabolism, leading to the production of some dopamine metabolites, such as dopamine quinone species. DA quinone species are demonstrated to be implicated in PD pathogenesis. Also, in cerebrospinal fluid and striatum of PD patients, the level of pro-inflammatory factors and anti-inflammatory cytokine (IL-4) is increased, demonstrating the connection between inflammation and PD [4].

Conventional therapies are intended to increase the dopamine level in the striatum. Levodopa, dopamine agonist, Monoamine Oxidase Inhibitors (MAO-I), and anticholinergic drugs are the main classes of drugs used to relief motoric symptoms. Although conventional drugs have substantially improved the quality of life in patients with PD, their correlated side effects (such as hallucination, involuntary movement, and limited long-term efficacy have indicated the need for alternative therapeutic strategies [5,6]. Under these circumstances, compounds derived from medicinal plants defined as plants containing substances used for therapeutic purposes or as precursors for drug synthesis have emerged as promising alternatives, offering potential neuroprotective benefits with a more favorable safety profile [7,8].

*Curcuma longa*, *Gastrodia elata*, *Ginkgo biloba*, *Paeonia alba radix*, *Pueraria lobata*, *Scutellaria baicalensis*, and *Withania somnifera* are some of the plants that have been investigated for their neuroprotective effects in PD models. *Gastrodia elata* reduces neuroinflammation, apoptosis, and motor deficits in PD models. *Ginkgo biloba* enhances dopamine levels and protects dopaminergic neurons. *Curcuma longa* preserves dopaminergic neurons, making it a potential candidate for PD therapy. Paeonia alba radix shows anti-inflammatory and autophagy-modulating properties. *Pueraria lobata* offers neuroprotection and reduces motor symptoms. *Scutellaria baicalensis* reduces oxidative stress and suppresses inflammation. Lastly, *Withania somnifera* enhances dopamine metabolism, contributing to its multifaceted neuroprotective actions.

This article aims to present a semi-systematic review of selected traditional herbs with neuroprotective properties, most of which are used in traditional Chinese medicine, specifically *Curcuma longa*, *Gastrodia elata*, *Ginkgo biloba*, *Paeonia alba radix*, *Pueraria lobata*, *Scutellaria baicalensis*, and *Withania somnifera*. These plants have demonstrated neuroprotective effects in neurodegenerative disorders, particularly PD, highlighting their potential as therapeutic alternatives in the future management of the disease. To our knowledge, no semi-systematic review has yet been conducted evaluating the neuroprotective roles of all these seven plants in PD. This underscores the need for a systematic synthesis of evidence on these plants to better understand their mechanisms in neuroprotection.

## 2. Methods

### 2.1. Protocol Registration

This semi-systematic review was registered on the Open Science Framework (OSF) https://osf.io/uxwhb (accessed on 26 July 2025), DOI 10.17605/OSF.IO/UXWHB.

### 2.2. Search Strategy

A comprehensive analysis of the literature available on PubMed, Scopus, and Web of Science between January 2020 to December 2024 was conducted on the potential role of seven medicinal plants: *Gastrodia elata blume*, *Ginkgo biloba*, *Curcuma longa*, *Pueraria lobata*, *Paeonia alba radix*, *Scutellaria baicalensis*, and *Withania somnifera* in PD.

Search terms included a combination of keywords (“Parkinson Disease”[MeSH Terms] OR “Parkinson’s disease” OR “PD”) AND (“Herbal Medicine”[MeSH Terms] OR “medicinal plants” OR “phytotherapy” OR “natural products”) AND (“*Pueraria lobata*” OR “Paeoniae Alba Radix” OR “*Ginkgo biloba*” OR “*Curcuma longa*” OR “*Withania somnifera*” OR “*Gastrodia elata blume*” OR “*Scutellaria baicalensis*”).

### 2.3. Inclusion and Exclusion Criteria

The inclusion criteria combined original research (in vitro, in vivo, or clinical trials), including case control, observational studies, case reports, or series. Narrative reviews and systematic reviews were also included. Editorials, opinions, abstracts, and posters were excluded. Only English language articles published between January 2020 and December 2024 were considered.

### 2.4. Study Selection

From the PubMed, Scopus, and Web of Science searches, a total of 161 articles were initially retrieved. These articles were imported into Rayyan for screening. During this process, 31 duplicates were identified and removed. As a result, 130 articles were retained for title and abstract screening, which were conducted independently by two reviewers (H.H. and I.Z.). Disagreements were resolved through discussion and consensus. In cases where consensus could not be reached, a third reviewer was consulted (M.H.). Only 17 articles were assessed for eligibility and included in the final review.

### 2.5. Data Extraction

To ensure accuracy and reduce bias, two reviewers independently conducted the data extraction process. Different variables, such as name of the medicinal plant, active compounds, cell line used, inducing agent(s) and its/their respective concentration, neuroprotective effects, experimental animal model used, route of administration, mechanism of action, blood–brainbarrier, and synuclein based model, are reported in Table 1 and Table 2, facilitating the comparison across studies and detecting recurring mechanisms involved in PD neuroprotection.

### 2.6. Search Results

**Figure 1:**
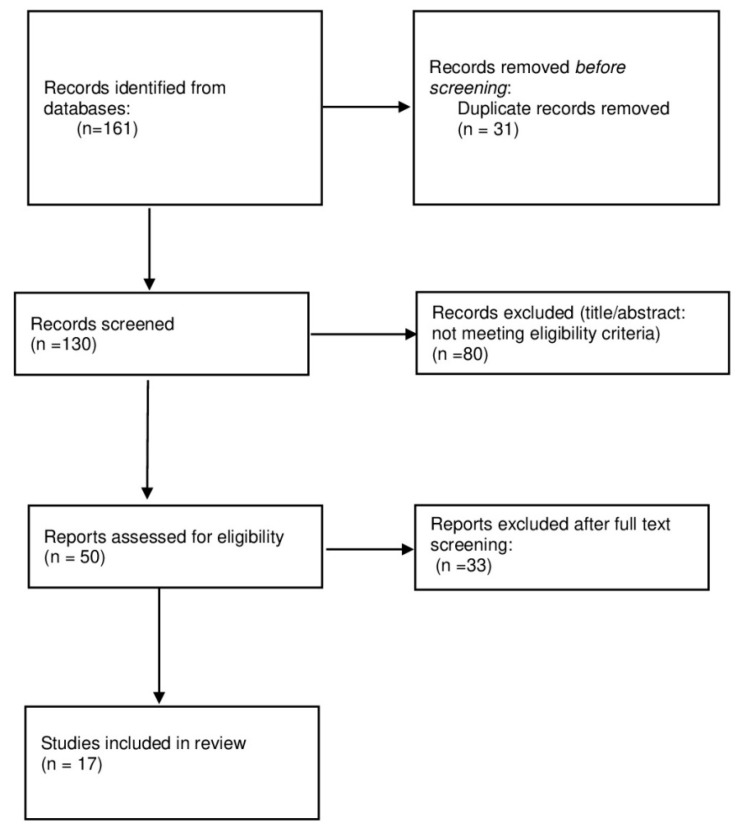
Semi-systematic review protocol as a flowchart.

The semi-systematic review protocol flowchart (Figure 1) recaps the study selection process, and the number of articles screened, excluded, and included in the final synthesis.

## 3. Results

### 3.1. Herbal Products and Parkinson’s Disease

Out of 161 articles, only 17 met the inclusion criteria upon investigating the neuroprotective effects of the selected medicinal plants in PD animal’s model. The studies were conducted in various in vitro (such as SH-SY5Y cells, PC12 cells, and LPS-induced microglial) and in vivo models (6-hydroxydopamine (6-OHDA), rotenone, and MPTP mouse). The studies evaluated either isolated active compounds (e.g., curcumin, gastrodin, and paeoniflorin) or standardized extracts (e.g., EGb 761 from *Ginkgo biloba*), evaluating the respective mechanisms, such as the antioxidant activity, anti-inflammatory pathways, mitochondrial protection, and apoptosis inhibition.

Overall, the results highlight the involvement of different neuroprotective pathways (Table 1 and Table 2).

#### 3.1.1. *Gastrodia Elata*

Belonging to the family of *Orchidaceae*, *Gastrodia elata* is an herb that has been utilized as a common remedy in eastern nations for many years because of its medicinal qualities [21]. Gastrodin, 4-hydroxybenzaldehyde, vanillyl alcohol, and vanillin are the major components of *Gastrodia elata*. Being able to cross the BBB, these compounds reveal many biological activities, including antioxidant, anti-asthmatic, antibacterial, and antimutagenic [46]. Gastrodin pretreatment (30–60 µmol/L) in BV2 microglial cells reduced LPS-induced expression of iNOS, COX-2, TNF-α, and IL-1β, indicating a notable anti-inflammatory effect [9]. Several studies draw attention to *Gastrodia elata*’s promise against PD. *Gastrodia elata* (10–200 mg/mL) improved cell viability and reduced apoptosis, ROS, and Bax/Bcl-2 ratio in MPP^+^-treated SH-SY5Y cells. Similarly, vanillyl alcohol at 10–200 mg/mL showed protective effects in MN9D cells. Gastrodin (10–60 mg/kg p.o. for 15 days) also reduced bradykinesia and motor deficits in MPTP-treated mice [10,21].

**Figure 2:**
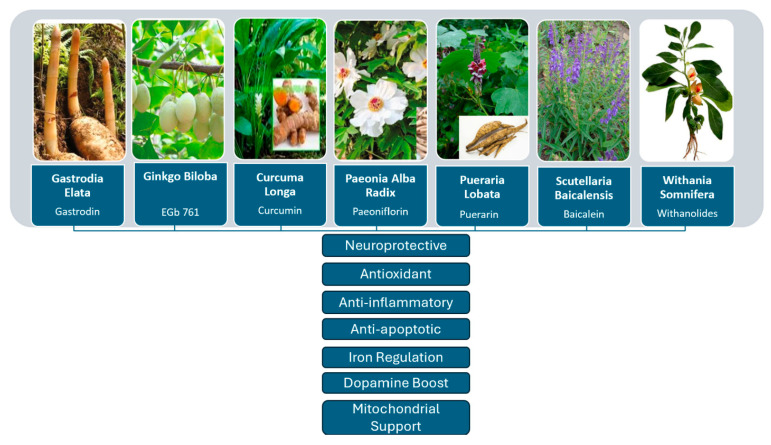
Schematic representation of the neuroprotective mechanisms of selected medicinal plants in Parkinson’s disease.

These findings suggest that the *Gastrodia elata* chemicals may be fit for clinical candidate development to reduce PD symptoms and have protective benefits in experimental PD models. The neuroprotective mechanisms of *Gastrodia elata* and other medicinal plants analyzed in this study are reported in Figure 2.

#### 3.1.2. *Ginkgo Biloba*

*Ginkgo biloba* (GB), a traditional Chinese herbal tree, is regarded as one of the oldest tree species in the world. Its extract has beneficial effects in the treatment of various diseases, including neurodegenerative disorders [10].

Different researchers have demonstrated that its extract, or EGB 761, has a positive effect on PD’s animal models, providing neuroprotection [47].

According to a study, in a 6-OHDA-induced Parkinsonian rat model, EGB 761 reduced behavioral abnormalities. It also improved locomotor activity, muscular coordination, and behavioral rotation when used as a supplement [22]. In those PD models, it also reduced the neurotoxicity caused by levodopa [12]. It was found that the use of GB supplements significantly increased the amount of dopamine and its metabolites [13].

Another study indicated that GB minimizes oxidative stress and cellular apoptosis in MPTP-induced PD models by boosting dopamine and antioxidant enzyme activity, while decreasing MDA levels. Furthermore, its use in a serpine-induced PD model contributed to the stabilization of redox balance, enhancement of mitochondrial activity and energy generation, and a reduction in apoptosis [14].

EGb 761 improves the proliferation of NSCs in the subventricular area in PD animals [48,49,50,51].

Another active compound, Ginkgetin, investigated in experimental PD models triggered by MPP^+^, under both in vitro and in vivo conditions, lowers intracellular reactive oxygen species, protects mitochondrial membrane potential, blocks apoptosis via the caspase-3 and Bcl-2/Bax pathway, and modulates iron metabolism [9].

Two other components, Ginkgolide B and bilobalide, have enhanced cell survival, reduced neuronal death, boosted locomotor activity, and restored dopaminergic markersin α-synuclein-expressing SH-SY5Y cells and A53T transgenic mice [12].

In vitro investigations of ginkgolic acid demonstrate a decrease in α-synuclein aggregation and SUMO-1 expression, accompanied with enhanced autophagosome production [13].

Protocatechuic acid amplifies the therapeutic efficacy of ginkgolide B, endorsing a synergistic approach for PD treatment [14]. In addition, in a randomized, double-blind placebo-controlled clinical trial, was noticed that 120 mg of Ginko Biloba twice daily, was not effective in reducing the overall incidence rate of dementia or AD incidence in elderly patients [48,49].

#### 3.1.3. *Curcuma Longa*

*Curcuma longa* L. (Cl), usually known as turmeric, is a native plant of Southeast Asia belonging to the Zingiberaceae family; it has therapeutic effects in PD and Alzheimer’s disease (AD). Turmeric has shown effects as an antioxidant, antimicrobial, anti-inflammatory, antibacterial, and anticarcinogenic agent [50].

Its polyphenolic constituents, especially curcuminoids, namely curcumin, bisdemethoxy curcumin, and desmethoxy curcumin, are responsible for pharmaceutical properties of *Curcuma longa* [51].

Studies have documented numerous bioactivities of curcumin, the main bioactive compound, including antioxidant, anti-inflammatory, antiproliferative, pro-apoptotic, antiparasitic, and antimalarial effects [52]. Curcumin exhibits antioxidant effects, boosts striatal dopamine, and protects dopaminergic neurons from oxidative damage in PD models.

A PD investigation found that curcumin, at concentrations less than 5 mmol/L, inhibited caspase-3 activation, reduced α-synuclein-induced intracellular ROS generation, and improved apoptotic signals in SH-SY5Y neuroblastoma cells [15].

Furthermore, following a curcumin pretreatment, PQ-exposed PINK1 siRNA cells demonstrated improved mitochondrial activity, decreased respiration, ATP generation, and apoptosis [16].

In an in vivo rat model of PD induced by 6-OHDA, curcumin has been shown to protect neurons from damage in the substantia nigra region of the brain. Curcumin administered intraperitoneally at 200 mg/kg was shown to mitigate the loss of tyrosine hydroxylase (TH) immunoreactive neurons in the substantia nigra, and striatal tyrosine hydroxylase (TH) fibers in mice treated with 6-OHDA [30].

Curcumin’s neuroprotective effects in Parkinson’s models are linked to reduced astroglial and microglial activation, likely due to its anti-inflammatory properties, along with decreased MDA levels and increased SOD, GPx, dopamine, and acetylcholine.

Curcuminoids’ neuroprotective properties were also tested by studies using MPTP-induced mouse models of PD. Oral preliminary treatment with curcuminoids at 150 mg/kg/day drastically reduced the production of total nitrite, cytokines, and protein inflammatory markers in MPTP-intoxicated mice. It also prevented dopamine depletion and loss of TH-positive neurons [31].

Curcumin (50 mg/kg i.p. daily) reduced dopaminergic axon loss and prevented neuron apoptosis in MPTP-treated mice [32]. Moreover, a synthetic curcumin derivative, curcuminglucoside, can bound with the oligomeric version of α-synuclein. This complex prevents the protein from further fibrillating. Its minimal toxicity is another advantage of using curcumin for PD.

A clinical study in AD suggests that curcumin decreases oxidative damage, removes stimulus for Aβ formation, reduces inflammation in microglial cells by lowering cytokine levels, and prevents Aβ plaque formation. It also triggers an enzyme that reduces the risk of Aβ production and lowers the oxidation of dangerous fats and proteins [53]. Therefore, based on the results mentioned above, it seems that Cl holds a lot of potential as a promising agent in PD clinical trials.

#### 3.1.4. *Paeonia Alba Radix*

The white root of Paeonia lactiflora Pall. (Paeoniaceae family), Paeoniae alba Radix, is often utilized as a bioactive compound of conventional Chinese remedies to target several issues [33].

The primary bioactive ingredient of *Paeonia alba radix*, paeoniflorin, has shown several pharmacological properties, including anti-allergic, anti-inflammatory, anti-hyperglycemic, analgesic, cognitive enhancement, antioxidant, etc. [34].

Curcumin (50 mg/kg i.p.), when taken daily, preserved dopaminergic axons and prevented neuronal apoptosis in MPTP mice. After therapy, 5 mg/kg of paeoniflorin considerably decreased neuroinflammation [33] and ameliorated dopaminergic neurodegeneration.

Paeoniflorin (2.5–10 mg/kg s.c., twice daily for 11 days) reduced 6-OHDA-induced damage in rats, likely via non-dopaminergic mechanisms, as it showed no direct action on D1 or D2 receptors [34].

Paeoniflorin is a potential substance for modulating autophagic activity and having neuroprotective effects [17]. According to a recent study, 50 mmol/L of paeoniflorin was shown to reduce the release of lactate dehydrogenase and the apoptotic rate, while also protecting PC12 cells from damage caused by MPP^+^ and acid through an autophagic pathway. Paeoniflorin (50 μmol/L) reduced α-synuclein toxicity in PC12 cells by enhancing its autophagic degradation via modulation of acid-sensing ion channels [18].

#### 3.1.5. *Pueraria Lobata*

*Pueraria lobata*, which has shown significant neuroprotective properties in relation to PD, has an active isoflavone molecule called puerarin, which has been demonstrated to alleviate motor symptoms and stop dopaminergic neuronal death, as well as dopamine depletion, in an MPTP-induced mice model of PD. Puerarin’s neuroprotective properties are credited to its capacity to increase GSH activity and activate the PI3K/Akt signaling pathway, therefore reducing oxidative stress while aiding neuronal survival. Moreover, puerarin is possibly involved in enhancing autophagy processes and lysosomal function. These results suggest that puerarin from *Pueraria lobata* might be a promising natural compound for the prevention and therapy of neurodegeneration in PD [35,36].

#### 3.1.6. *Scutellaria Baicalensis*

In Chinese medicine, *Scutellaria baicalensis* (SB), a plant belonging to the Lamiaceae family, is a common and traditional medicinal herb. Flavonoids, including baicalein, baicalin, scutellarin, and wogonin, are the principal bioactive ingredients of SB, which have been well demonstrated to have anti-inflammatory [54], antioxidant [55], and neuroprotective properties [56]. Several studies have proved that extracts of SB or its separated components have notable neuroprotective effects in different models of neurodegenerative diseases [57,58,59]. Because of its many pharmacological properties, this plant has attracted more and more interest for possible use in the management of PD.

One of the pathogenic factors of PD is oxidative stress (OS). The substantia nigra is particularly vulnerable to OS, and, as a result, it puts PD patients at risk of neurodegenerative disorders [60]. Consequently, it is more frequent for older adults to be diagnosed with PD in clinical evaluation.

Malondialdehyde (MDA) levels rise in PD animal models subjected to oxidative stress, although antioxidant enzyme activity, including that of GSH-Px, CAT, and SOD, is markedly decreased. The rats are protected by baicalin from the oxidative damage caused by 6-OHDA. Furthermore, in PD model rats [37], after baicalin treatment, monoamine neurotransmitter release was raised, and abnormal behavioral signs, such stiffness and body tremors, were improved. Baicalein showed neuroprotection by reducing nuclear damage and cell death in rotenone-treated cells. Baicalein significantly reduced ROS, apoptosis, and PD symptoms, while baicalin lowered MDA and boosted GSH and GSH-Px levels in MPTP-induced PD mice [19,38].

Baicalein lowered cleaved caspase-3 protein levels and reduced OS in vivo in a 6-OHDA-treated model of Cryptococcus hippocastanum infection by lowering MDA levels [39].

Recently researchers have revealed a connection between baicalin’s neuroprotective action in PD models and the Nrf2 pathway. Baicalin reduced oxidative stress, activation of microglial cells, and inflammatory reactions by means of substantial Nrf2 and downstream antioxidant enzyme expression upregulation and NLRP3 inflammatory vesicle activation suppression [61].

Moreover, the brain has been noted to have an irregular distribution of Fe^2+^. Many studies have shown a link between iron deposition and the expression of PD-related symptoms. The substantia nigra has relatively high Fe^2+^ levels concentrated in its thick zone [62]. DA neurons are hence impacted from Fenton or Haber–Weiss reactions. These mechanisms convert H_2_O_2_ to OH, hence increasing OS, causing DNA damage, and causing autophagic cell death [63]. Studies emphasize the increased OS marker and iron buildup in brain tissue in PD patients.

Earlier studies showed that baicalein lowers iron levels in the substantia nigra via two main pathways: adversely influencing the expression of iron carrier protein 1 (FP1) and positively regulating the expression of divalent metallic carrier protein 1 (DMT1) [64]. Another PD study finds that baicalin lowers MDA levels, raises GSH levels, and controls DMT1 and FP1 expression, thereby reducing iron buildup and OS levels [65]. Reducing iron buildup in the substantia nigra might help baicalin to relieve oxidative stress and alleviate PD symptoms.

On the other hand, clinical data on baicalin and its glycoside baicalein have received attention regarding their safety and adverse effects. A randomized, double-blind experiment involving 72 healthy individuals found no negative side effects when a single oral dose of baicalin and baicalein, ranging from 100 to 2800 mg, was administered. No evidence of hepatic or renal toxicity was observed in the laboratory assessments conducted during this study [66]

In a safety study, 36 healthy individuals received 200–600 mg of oral baicalein, with only mild, self-limiting side effects reported [67].

This does not mean that baicalin and baicalein are entirely safe. There are some trial results which have shown that some participants experienced adverse events, such as an increase in high-sensitivity C-reactive protein (hs-CRP) and triglyceride levels, while on baicalein tablets [68]. Yi Cai et al. reported that doses of 800–1600 mg/kg for 8 weeks could pose a risk of nephrotoxicity in rats [69].

The oral administration of baicalein has been shown to be safe for humans during phase I/II of clinical trials [70]. However, further investigation into baicalein’s therapeutic potential in patients with various diseases is required.

#### 3.1.7. *Withania Somnifera*

One of the Nigerian medicinal herbs, *Withania somnifera* (Ws) (known as Karamanta in Hausa), is used to treat several illnesses, including PD, stress, arthritis, and other issues relating to the central nervous system [40,71]. Prakash and his team investigated how Ws affected dopamine and its metabolites in the central nervous system (SNpc) area of PD animals. Dopamine and its derivatives in the PD mice brain were shown to be less concentrated than in the control. In contrast, dopamine and its metabolites (DOPAC and HVA) were significantly elevated after nine weeks of Ws treatment. This supports the assertion that Ws can increase the levels of catecholamine and combat PD-like conditions [41,42]. The improvement of mitochondrial and endothelial activity, apoptotic reduction, anti-inflammatory actions, and control of oxidative stress are the main mechanisms of action by which Ws shows its neuroprotective effects [40,43]. MPTP induced Parkinson-like symptoms in both Balb/c mice [42,44] and 6-OHDA PD models [40,45]. Ethanolic extract of Ws has significantly reduced the oxidative stress profile, alongside a decrease in motor function.

KSM-66, a *Withania somnifera* root extract, showed neuroprotective effects against 6-OHDA-induced toxicity in SH-SY5Y cells by modulating oxidative stress and redox regulation, particularly S-glutathionylation, when administered before or after toxin exposure. Before and after 6-OHDA cell treatment, KSM-66 doses ranging from 0.25 to 1 mg/mL were used. The extract greatly improved thioltransferase activity and glutathione peroxidase activity after pre- or post-6-OHDA treatment. Furthermore, S-glutathionylation allowed the extract to modulate redox control. Protein-glutathionylation levels in 6-OHDA-treated cells were lower in KSM-66’s pretreatment of SH-SY5Y cells. Higher ATP levels were seen with the restoration of mitochondria using 0.5 mg/mL of KSM-66 extract [20].

Ws’s neuroprotective action might be credited to its bioactive chemical components, including alkaloids, which would be a possible target for developing innovative therapies for PD [20,72].

## 4. Discussion

In recent years, more than a hundred publications have explored the potential of various natural products and herbs in treating PD. This study spotlights the role of some neuroprotective traditional herbs, such as *Curcuma longa*, *Gastrodia elata blume*, *Ginkgo biloba*, *Paeonia alba radix*, *Pueraria lobata*, *Scutellaria baicalensis*, and *Withania somnifera*, with potential benefits in managing PD symptoms and elucidates their specific mechanisms of action.

We decided to use these plants mainly due to their neuroprotective role, as supported by preclinical and animal evidence, and the use of the majority of them in traditional Chinese medicine. However, it is important to note that these herbs are not considered primary treatments for Parkinson’s disease, but rather are used as preventive or adjunctive agents to support conventional therapies and potentially enhance neuroprotection. Based on a comparative analysis of the studies reviewed, only two medicinal plants, *Curcuma longa* and *Ginkgo biloba*, have been studied more extensively in the context of PD. This is reflected in the variety of experimental models, active compounds examined, and identified mechanisms of action. Multiple preclinical models (e.g., SH-SY5Y cells, MPTP mice, and 6-OHDA rats) have confirmed a wide range of neuroprotective mechanisms of action of curcumin, the main active compound of *Curcuma longa*. Early efforts toward pharmaceutical development have been made by producing semi-synthetic derivates such as curcuminglucoside, a promising agent in stabilizing α-synuclein. Laterally, *Ginkgo biloba* is on an encouraging path for drug development, as a high quantity of studies have been conducted on its compounds. EGb 761 has experienced multiple stages of development and numerous biopharmaceutical analysis. This is not the case for plants such as *Gastrodia elata blume*, *Paeonia alba radix*, *Pueraria lobata*, *Scutellaria baicalensis*, and *Withania somnifera*, which are promising but have a more limited and less standardized study base.

While the pharmacological properties of these medicinal plants have been investigated in different studies in vitro and in vivo, their safety and potential adverse effects and clinical evidence in humans have also come into focus, as well as their safety profiles during pregnancy and lactation.

Although the neuroprotective potential of these medicinal plants has been demonstrated in cellular and animal models, there is a significant gap between experimental data and clinical adaptation, because none of the selected plants has been studied on a large scale or in PD populations. Some of them are used in clinical trials in other diseases, as mentioned previously.

Based on the sources, there is no direct information regarding the safety or effects of these medicinal plants and their compounds during human pregnancy or lactation. Reproductive toxicity and perinatal safety have not been assessed for the bioactive compounds of these plants, such as withanolides, curcumin, and baicalin.

Although these findings demonstrate the neuroprotective effect of these medicinal plants by different mechanisms of action, such as antioxidative stress mitigation, anti-inflammatory responses, and support of mitochondrial function, limitations in standardization, understanding pharmacokinetics, and reproducibility across studies, as well as absence of extended clinical trials, restrict their application in PD treatment protocols.

One limitation of this study is that it focuses on only seven plants with reported neuroprotective effects, while many other medicinal plants with potential benefits in PD models remain unexamined. This introduces a potential selection bias. For example, plants such as *Mucuna pruriens*, *Vicia faba*, *Nigella sativa*, *Amomum tsao-ko*, *Trifolium pratense*, *Glycine max*, and *Crocus sativus* have also been investigated for their neuroprotective properties. These species have demonstrated beneficial actions, including antioxidant activity, modulation of dopamine levels, and protection of dopaminergic neurons, as supported by both preclinical and traditional medicine studies [73,74,75,76]. Therefore, future reviews incorporating a broader range of plants are warranted to fully capture the therapeutic potential of medicinal plants in PD.

## 5. Conclusions and Future Perspectives

This review underlines the promising role of medicinal plants in decelerating the progression of PD through different mechanisms, such as antioxidant activity, anti-inflammatory effects, and mitochondrial protection. The active compounds of the plants mentioned in this semi-systematic review have been demonstrated to be valuable in preclinical models, thus supporting their use as alternative treatments.

These natural compounds not only offer symptomatic relief in PD, but also contribute to slowing neurodegenerative progression of the disease by different mechanisms of action, including restorative properties of neurons (e.g., withanolides from *Withania somnifera*), inhibition of pro-inflammatory mediators (e.g., gastrodin from *Gastrodia elata*), and preservation of mitochondrial function (e.g., ginkgolide from *Ginkgo biloba*).

While recent studies have demonstrated reliable outcomes for these medicinal plants, there is a significant gap between the experimental evidence and clinical application. Limited data on their bioavailability, long-term safety profiles, interaction with other drugs, and poor data from high-quality randomized controlled trials are the main issues to be pointed out in this gap. Also, a lack of standardized formulations can lead to inconsistent results across studies, limiting the reproducibility.

Future perspectives should focus on interdisciplinary collaboration between pharmacologists, neurologists, and traditional medicine researchers on conducting well-designed randomized clinical trials to validate the transition from experimental promise to therapeutic application.

## Figures and Tables

**Table 1:** Summary of medicinal plants used in cellular models of PD.

Medicinal Plant	Active Compounds	Cell Line Used	Inducing Agent	Plant Concentration	Neuroprotective Effect	Ref.
*Gastrodia elata*	Gastrodin, vanillyl alcohol, vanillin	Murine microglial BV 2 cells; SH-SY5Y cells; Dopaminergic MN9D cells	MPP^+^	30, 40, and 60 mmol/L; 10 to 200 mg/mL; 10 to 200 mg/mL	Anti-inflammatory; ↓apoptosis; ↓ROS; ↓Bax/Bcl-2 ratio; ↑ cell viability	[9,10]
*Ginkgo biloba*	EGb 761 (standardized extract), flavonoids, terpenoids	SH-SY5Y cells	α-synuclein	Not specified	↓ ROS; modulated iron metabolism; blocked apoptosis	[11,12,13,14]
*Curcuma longa*	Curcumin, bisdemethoxycurcumin, desmethoxycurcumin	SH-SY5Y cells; PINK1 siRNA cells	α-synuclein; paraquat	<5 mmol/L; not specified	Blocks apoptosis and caspase 3 activation; Improve mitochondrial activity	[15,16]
*Paeonia alba radix*	Paeoniflorin	PC12 cells	MPP^+^	50 mmol/L	↓ Apoptosis; ↓release of lactate dehydrogenase	[17,18]
*Pueraria lobata*	Puerarin	Not reported	Not reported	Not reported	Not reported	N.A
*Scutellaria baicalensis*	Baicalein, baicalin	Not reported	Rotenone	Not specified	↓ Apoptosis; ↓ ROS	[19]
*Withania somnifera*	Withanolides (withaferin A, withanolide D)	SH-SY5Y cells	6-OHDA	0.25 to 1 mg/mL	↑ (GPx) activity; ↓ S-glutathionylation; Improves mitochondrial activity; Modulates oxidative stress proteins	[20]

Legend: N.A., not applicable; no eligible study was identified.

**Table 2:** Summary of medicinal plants used in animal models of PD.

Medicinal Plant	Active Compounds	Animal Studies	Inducing Agent	Plant Concentration	Neuroprotective Effect	Route of Administration	BBB Permeability	Synuclein-Based Model	Ref.
*Gastrodia elata*	Gastrodin, vanillyl alcohol, vanillin	Mice	MPTP	10, 30, and 60 mg/kg	↓Bradykinesia and motor dysfunction	Oral	Yes	Not specified	[21]
*Ginkgo biloba*	EGb 761 (standardized extract), flavonoids, terpenoids	Rats; mice; serpine	6-OHDA; MPTP; MPTP; α-synuclein	Not specified	Improves motor coordination; ↓ levodopa toxicity; ↓ apoptosis and oxidative stress; ↑ dopamine and metabolites; ↑ cell survival and↓ neuronal death.	Not specified	Not specified	Yes	[11,22,23,24,25,26,27,28,29]
*Curcuma longa*	Curcumin, bisdemethoxycurcumin, desmethoxycurcumin	Rats; mice	6-OHDA; MPTP	200 mg/kg; 150 mg/kg/day; 50 mg/kg/day	↑ Dopamine; ↓ GSH depletion, protein oxidation, inflammation, and apoptosis; protects nigral neurons; ↓astroglial/microglial activation, MDA; ↑ SOD, GPx, and Ach	Intraperitoneal; oral	Not specified	Not specified	[30,31,32]
*Paeonia alba radix*	Paeoniflorin	Mice; rats	MPTP; 6-OHDA	2.5 and 5 mg/kg; 2.5, 5 and 10 mg/kg twice a day	Protects striatal neurons; ↓ bradykinesia, neuroinflammation; improves dopaminergic survival; may modulate autophagy	Subcutaneous	Not specified	Not specified	[33,34]
*Pueraria lobata*	Puerarin	Mice	MPTP	Not specified	Improves motor symptoms and dopamine loss; ↑ GSH, GDNF; activates PI3K/Akt; reduces oxidative stress; restores Lamp-2A expression and autophagy	Not specified	Not specified	Not specified	[35,36]
*Scutellariabaicalensis*	Baicalein, baicalin	Rats; mice; Cryptococcus hippocastanum	6-OHDA; MPTP; 6-OHDA	Not specified	↓ Oxidative damage and motor deficits; ↑ neurotransmitter release; ↓ MDA, caspase-3, iron accumulation; ↑ GSH, GPx, SOD, CAT, GR, Nrf2, and antioxidant enzymes	Not specified	Not specified	Not specified	[37,38,39]
*Withaniasomnifera*	Withanolides (withaferin A, withanolide D)	Mice	MPTP; 6-OHDA	Not specified	↑ Dopamine, DOPAC, HVA, mitochondrial and endothelial function; ↓ apoptosis, inflammation, oxidative stress; and improves motor function	Not specified	Not specified	Not specified	[40,41,42,43,44,45]

Legend: BBB (blood–brainbarrier).

## Abbreviations

6-OHDA	6-Hydroxydopamine
BBB	Blood–brain barrier
CNS	Central nervous system
DA	Dopamine
ETC	Electron Transport Chain
GABA	Gamma-Aminobutyric Acid
GPi	Globus Pallidus Internus
GSH	Glutathione
hs-CRP	High-sensitivity C-reactive protein
IL-1β	Interleukin-1 beta
IL-4	Interleukin-4
IL-6	Interleukin-6
MAO-I	Monoamine Oxidase Inhibitors
MDA	Malondialdehyde
MPTP	1-Methyl-4-phenyl-1, 2, 3, 6-tetrahydropyridine
OS	Oxidative stress
PD	Parkinson’s disease
p.o	Per Os
ROS	Reactive oxygen species
SNr	Substantia nigra pars reticulata
TNF-α	Tumor necrosis factoralpha
